# Ecotype Differentiation in the Face of Gene Flow within the Diving Beetle *Agabus bipustulatus* (Linnaeus, 1767) in Northern Scandinavia

**DOI:** 10.1371/journal.pone.0031381

**Published:** 2012-02-13

**Authors:** Marcus K. Drotz, Tomas Brodin, Anssi Saura, Barbara E. Giles

**Affiliations:** 1 Lake Vänern Museum of Natural and Cultural History, Lidköping, Sweden; 2 Department of Ecology and Environmental Science, Umeå University, Umeå, Sweden; 3 Department of Molecular Biology, Umeå University, Umeå, Sweden; Instituto de Higiene e Medicina Tropical, Portugal

## Abstract

The repeated occurrence of habitat-specific polyphyletic evolved ecotypes throughout the ranges of widely distributed species implies that multiple, independent and parallel selection events have taken place. Ecological transitions across altitudinal gradients over short geographical distances are often associated with variation in habitat-related fitness, these patterns suggest the action of strong selective forces. Genetic markers will therefore contribute differently to differences between ecotypes in local hybrid zones. Here we have studied the adaptive divergence between ecotypes of the water beetle *Agabus bipustulatus* along several parallel altitudinal gradients in northern Scandinavia. This water beetle is well known for its remarkable morphological variation associated with mountain regions throughout the western Palaearctic. Two morphological ecotypes are recognised: a montane type with reduced flight muscles and a lowland type with fully developed muscles. Using a multilocus survey of allozyme variation and a morphological analysis with landmark-based morphometrics, across thirty-three populations and seven altitudinal gradients, we studied the local adaptive process of gene flow and selection in detail. Populations were sampled at three different elevations: below, at and above the tree line. The results indicate that the levels of divergence observed between ecotypes in morphology and allele frequencies at α-*Glycerophosphate dehydrogenase* relative to those shown by neutral molecular markers reflects local diversifying selection *in situ*. Four main lines of evidence are shown here: (1) A repeated morphological pattern of differentiation is observed across all altitudinal transects, with high reclassification probabilities. (2) Allele and genotype frequencies at the *α-Gpdh* locus are strongly correlated with altitude, in sharp contrast to the presumable neutral markers. (3) Genetic differentiation is two to three times higher among populations across the tree line than among populations at or below. (4) Genetic differentiation between ecotypes within independent mountain areas is reflected by different sets of allozymes.

## Introduction

Gene flow is traditionally seen as the key factor that holds gene pools of local populations together, homogenising the genetic variation of interbreeding populations and opposing the effects of drift and local selection [1]–[2]. Recent empirical evidence challenges the universality of this view; site-specific selection and non-random mating have been shown to cause strong differentiation and/or reproductive isolation over small geographical scales [3]–[7]. As a result, focus is shifting towards understanding the evolutionary mechanisms driving adaptive versus neutral genetic differentiation within species where ecotypes exhibit sharp phenotypic differences across environmental boundaries over distances well within the dispersal capacities of a species [8]–[13]. The repeated occurrence of habitat-specific ecotypes within a widespread species is now taken as evidence for adaptation and, where gene flow can occur between ecotypes, that the process of divergence is driven by strong natural selection [5], [14]–[16]. Ultimately this knowledge will help to resolve one of the more contentious issues in evolutionary biology on how species or ecotypes may evolve and diverge in the absence of geographical barriers, namely, sympatric speciation. To date few credible examples of this process have been demonstrated although the number is growing [11], [16]–[18].

**Figure 1:**
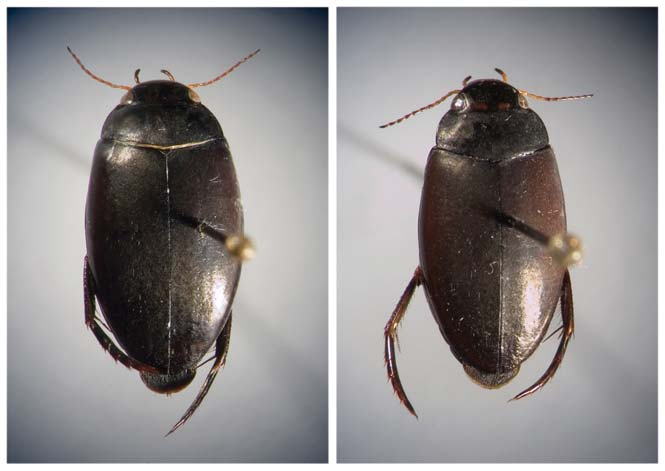
The two ecotypes of *Agabus bipustulatus* in northern Sweden: lowland (left side) and montane (right side).

In general, ideal systems for exploring the potential role of diversifying selection for ecotypic divergence are found where common environmental factors, varying across some gradient, repeatedly lead to the existence of similar phenotypic forms in independent geographic localities [3], [12]–[13]. In this study, we focus on the water beetle *Agabus bipustulatus* (Linnaeus, 1767) one of the most common water beetles in the western Palearctic. It shows a remarkable morphological and geographical variation strongly associated with high altitude mountain regions [19]–[23]. Two morphologically distinct ecotypes are recognised [19], [24] (Fig. 1): a montane type with reduced flight muscles and a lowland type with fully developed flight muscles [25]–[28]. The lowland form is known to be a strong flier able to migrate over the distances typically separating populations below the tree line, above the tree line and between mountains see [26]–[27], [29]–[30]. In contrast, the reduced flight muscles of the montane form severely limit its dispersal capacity [28]. This form may migrate passively via downstream drift between populations within the same watercourse but migration between mountain tops is highly unlikely in the absence of flight. In addition to these disjunct distributions of morphological forms/ecotypes, the *α-Glycerophosphate dehydrogenase* locus (*α-Gpdh*), a major regulating enzyme in the metabolic pathway generating energy for flight [31]–[33], has been shown to display strong divergence between populations in the lowland areas and the alpine zones above the tree line. The locus has thus been identified as a candidate locus subject to selection mediated by factor(s) related to altitude and climate [21]. A phylogeographic analysis of the *A. bipustulatus* complex also confirmed that the associations between the *α-Gpdh* genotypes and altitude are observed across the entire west Palaearctic region [22] and that the montane ecotype has polyphyletic origins across the region [23].

These repeated and parallel distributions of morphological and enzyme phenotypes make *A. bipustulatus* an ideal system to study local environmental adaptation in the absence of gene barriers [12]. The Scandes mountain range provides an ecological theatre where these processes can be studied in more detail and on a scale where populations are more connected. Intermixed populations containing a few specimens of either ecotype have occasionally been observed above and below the tree line [28]. Looking across the Scandes mountain range, the distribution pattern of these ecotypes is, however, striking – populations on mountain peaks consist primarily of the montane form whereas all populations below the peaks consist almost entirely of the lowland type [19].

The purpose of this study was to analyse whether habitat-specific selection in *A. bipustulatus* is sufficiently strong to impede local gene flow across several independent parallel environmental gradients in Northern Scandinavia. Specifically, we analysed patterns of variation in *α-Gpdh* and morphological traits as well as in putatively neutral allozyme markers to assess genetic differentiation among populations across one longitudinal and seven altitudinal environmental gradients. These gradients span over three distinct climatic zones ranging from boreal through subalpine to alpine.

If *in situ* divergent selection is important for creating and maintaining ecotypic differentiation in the presence of countervailing gene flow in *A. bipustulatus*, we expect (i) sharp changes in morphological and *α-Gpdh* phenotypes, i.e. the traits subject to selection, across all tree lines. Since selectively neutral loci should show random patterns of variation among populations, especially given the potential for migration among populations, then if selection is imposing reproductive isolation (ii) the strongest differentiation in neutral markers should be observed between populations above and below the tree line both within and between mountains. Furthermore, individuals of different ecotypes within mountains should be less differentiated than individuals of the same ecotype from different mountains. (iii) If this is occurring independently in each location, the combination of neutral loci reflecting this differentiation should be specific to each mountain site.

## Materials and Methods

### The water beetle

*Agabus bipustulatus* is a dark medium-sized water beetle with a Palearctic distribution. The lowland ecotype is found in many types of habitats. In the boreal region, it prefers smaller streams, and springs and pools with little vegetation and stony bottoms. Above the tree line, the montane ecotype is most common in shallow lakes with no fish, chiefly with stony bottoms and some marginal vegetation [20].

The timing of male accessory gland enlargement indicates that the mating season is between May and September [34]. Eggs are laid either within submerged vegetation or in soft dry ground. Females generally carry one spermatophore, although two have been observed [35]. Females can lay between 600–800 eggs from June to August and have been observed to lay fertile eggs 12 months after the last copulation [34]–[35]. The eggs mature from early August to late April after which they hatch into first instar larvae. *Agabus bipustulatus* has three larval instars [36]–[37], all of which can be found almost year round in the lowlands of northern Sweden. In late autumn it is rare to find any first or second instar larvae above the tree line, where the third instar prevails [28]. After the third and final instar, the larvae enter the pupal stage which lasts for 42 days (at 11.3°C) [34]. Both adults and larvae are able to overwinter [20].

Water beetles have two major means of dispersal. For short-distance dispersal (i.e. within a pond/lake), they swim or, if dispersal occurs along running water, passively drift with the current. The second mean of dispersal, flying, is mainly used for longer distances (i.e. between ponds/lakes). Main period for long-distance dispersal occurs in late summer [20]. The capacity for flight is not equally important for all species of water beetles and they show variation in flight muscle development, from absent to fully developed [25]. The mechanism behind this within-species variation has not yet been resolved. One plausible reason could be that a partial to full histolysis of the flight muscle occurs when flight capacity is obsolete. However, a study by Jackson [25] shows that no beetles with abnormal flight muscles could be induced to fly and the development of flight muscles and their support appeared to be arrested at an early stage. Her study also indicated a positive relationship between flightless forms and colder environments. Although as far as we know no study has documented the genetic control of flight muscle development. Studies of dispersal distances in *A. bipustulatus* are scarce but have shown that lowland forms with fully developed flight muscles disperse over distances up to several hundred meters [30]. Low mark-recapture numbers in areas with ponds separated by less than a couple of hundred meters indicate that the beetles can perform longer flights [34], [38]. Other studies of similar sized water beetles have shown that they readily disperse several kilometres and distances of up to 25 km have been recorded [29]. Bilton [39] gives evidence for long-distance flying while Jackson [25] has tied flying ability to the development of the flight muscles. Low recapture rates with the studies cited above strengthen the idea that flight in the lowland form of *A. bipustulatus* is migratory as suggested by Southwood [40] and Dingle [41].

### Sample collection

**Figure 2:**
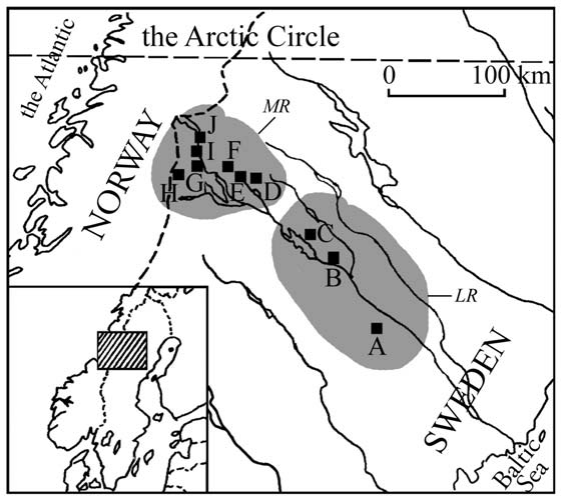
*Agabus bipustulatus* collecting areas A – J and their spatial relationship to the two main sample regions. The Lowland region (LR) and mountain region (MR) in the upper Ume river valley mountain region in northern Sweden are marked gray.

A total of 1279 individuals from 33 populations were sampled within seven altitudinal transects and one reference latitudinal transect in the Swedish Lapland during September and early October, 1998 (Fig. 2, Table 1). This sampling time is just after peak migration, which maximises the probability of finding both types of beetles in all habitats. Adult individuals were collected with a water bag net near rocks or overhanging vegetation at several locations along the margins of large water bodies, or throughout the entire water area in small water bodies. The specimens collected for the enzyme assays were kept alive and transported in damp moss to the laboratory where they were then separated by sex and frozen at −70°C. Specimens used in the morphological analyses were kept in 95% ethanol in a refrigerator at 4°C. Populations used in the morphological analyses are marked in italics in table 1.

**Table 1:** Population variables of the sampled Agabus bipustulatus populations in northern Sweden.

Area		Population	Altitude *(m asl)*	Elevation level	N	*F*_IP_	Genetic diversity	*α-Gpdh* 100/100 frequency	*α-Gpdh* 100/108 frequency
**A**	1	*Näslandsmyren*	300	LR	34±3	.324 **	.188±.069	0.33	0.42
	2	*Rörmyrberget-III*	440	LR	27±1	.270 ns	.275±.086	0.58	0.42
	3	Rörmyrberget-I	510	LR	21±2	.072 **	.212±.074	0.29	0.38
**B**	1	Grundfors	310	LR	8±1	.059 ns	.404±.112	0.25	0.38
	2	*Björkliden*	440	LR	12±1	.407 *	.178±.049	0.44	0.44
**C**	1	*Ersmark*	400	LR	22±2	−.032 ns	.259±.069	0.25	0.63
	2	Kyrkberget	480	LR	13±1	.113 ns	.364±.096	0.00	0.50
**D**	1	*Brånaberg-II*	480	MB	44±2	.432 *	.326±.080	0.43	0.29
	2	*Atjekberget*	570	MB	29±2	.310 *	.266±.067	0.54	0.23
	3	Unna Suojal	810	MT	24±2	.233 *	.272±.064	0.58	0.33
**E**	1	Kråkberget-IV	670	MT	8±1	.083 ns	.281±.100	0.00	1.00
	2	Kråkberget-V	670	MT	24±3	.287 *	.224±.066	0.50	0.50
	3	*Kråkberget-VI*	770	MT	33±3	.188 *	.287±.086	0.36	0.50
	4	*Kråkberget-VII*	800	MA	25±1	.140 *	.230±.078	0.36	0.55
**F**	1	Oltokholmen-IX	460	MB	12±1	.167 ns	.250±.069	0.33	0.50
	2	*Oltokholmen-VIII*	460	MB	14±2	.195 ns	.212±.077	0.27	0.67
	3	*Gäutavardo*	840	MA	15±1	.449 *	.281±.074	0.17	0.83
**G**	1	*Rundtjärn*	700	MT	21±2	.131 ns	.397±.089	0.00	0.88
	2	Västansjö-XVI	720	MT	12±1	.170 ns	.337±.069	0.20	0.80
	3	Västansjö-XVII	740	MT	6±1	.191 ns	.329±.059	0.50	0.50
	4	*Gieravardo*	770	MA	20±1	.056 **	.182±.057	0.50	0.48
**H**	1	*Gröndal-XX*	590	MB	13±1	.089 ns	.299±.088	0.50	0.50
	2	Gröndal-XXI	610	MB	5±1	.295 ns	.222±.092	0.50	0.50
	3	*Atoklinten-I*	800	MT	39±2	.219 *	.273±.056	0.55	0.39
	4	*Atoklinten-V*	840	MT	23±1	.349 **	.212±.057	0.86	0.07
	5	*Atoklinten-II*	870	MA	28±2	.342 **	.248±.071	0.97	0.00
	6	*Atoklinten-III*	920	MA	11±1	.030 ns	.257±.087	1.00	0.00
	7	*Atoklinten-IV*	930	MA	53±4	.313 **	.234±.063	0.89	0.11
**I**	1	Klippen-XII	480	MB	10±1	.230 ns	.188±.060	0.20	0.20
	2	Stintbäcken	520	MB	42±4	.115 **	.215±.066	0.25	0.54
	3	*Djuptjärn*	740	MA	75±4	.216 **	.227±.057	0.60	0.40
**J**	1	*Lillbraket-XIII*	675	MT	20±3	.114 ns	.280±.078	0.10	0.60
	2	*Braket-XIV*	730	MA	51±5	.156 **	.153±.048	0.50	0.26

### Sampling design

The single latitudinal transect begins c. 100 km to the east of the mountain region and runs eastward towards the Baltic Sea coast for c. 200 km. This lowland region (LR) transect contains three sampled areas (named A-C), of which A has three populations and B and C have two each (Fig. 2). The seven altitudinal transects within the mountain region (MR) are located within different mountain districts, hereafter named D-J. All districts except D are separated by at least 15 km and have a mean diameter of 1–6 km. Area D has a diameter of 15 km but does not overlap with any other area. Districts I and J share the same mountain valley. Each of the altitudinal transects are divided into three elevation zones for sample collecting; below the tree line (hereafter referred to MB), at the tree line (MT) and above the tree line (MA). The tree line is defined as the highest continuous altitudinal level of *Betula pubescens* ssp. *czerepanovii*. It constitutes a major ecosystem shift between boreal and alpine zones [42]–[43]. All populations sampled above the tree line are from large shallow tarns, except Djuptjärn (I3), which is a deep water-turbine shaft. Population sites at the tree line vary from small cold spring outlets (Lillbraket-XIII, J1) and larger tarns (i.e. Kråkberget-IV–V (E1–4) and Unna Suojal, D3) to a large drilled shaft hole (Rundtjärn, G1). The habitats below the tree line vary extensively, but most represent stable water bodies. The small number of specimens and the apparent instability of small shallow bodies of water with a high risk of drying-out, i.e. the habitats at Kråkberget-IV (E1), Västansjö-XVII (G3), Gröndal-XXI (H2) and Atoklinten-III (H6), suggest that these populations were newly founded.

### Morphological analyses

Geometric morphometrics utilise the spatial covariation of homologous landmarks to calculate affine (*partial warps*) and nonaffine (*uniform*) shape components. These components can be used to calculate differences between morphology within and between ecotypes or the associations between shape and other parameters (e.g. altitude or genetic variation at specific loci (here *α-Gpdh*)) [44]–[46]. The shape component is a decomposed description of one specimen in relation to a consensus shape of all specimens in the analysis. Each landmark (geometric position) of a specimen is scaled (centroid size = 1), rotated, translated and aligned to minimise differences between samples (230 specimens in total, Table 1). The Procrustes metric (α) was set to null [47], which gives equal weights to partial warps at all spatial scales. The uniform component estimated as described by Bookstein [48] was included in the weight matrix.

We used eight landmarks, representing the beetles' right side (Fig. 1): (1) Right elytron, posterior apex; (2) Right elytron, right anteriolateral angle; (3) Pronotum, right posteriolateral angle; (4) Pronotum, midpoint between landmarks 3 and 5; (5) Pronotum, right anteriolateral angle; (6) Pronotum, central anterior margin; (7) Pronotum, central posterior margin; (8) Right elytron, lateral point at widest portion. These landmarks were selected to provide coverage of the shape variation observed in pronotal length, width and its anteriolateral variation relative to the elytra between the ecotypes [19], [49]. Landmarks were not used on the head as its position relative to the rest of the body may obscure the analysis [50]–[51]. A Summa Sketch III (Summagraphics) graphics tablet was used to capture the geometric position of each landmark relative to a Cartesian coordinate. To assess the repeatability and minimise measurement errors of the geometric morphometric analyses, each specimen was measured three independent times as described by Lessells and Boag [52].

Morphological variation within the total sample was analysed with a Principle Component Analysis (PCA) of the partial warps and the uniform components in the Relative warps program v 1.20 [53]. The obtained components (*relative warps*) are only used to describe the shape variation and were not included in any statistical analysis [45]–[46]. The relationship between morphology (partial warp score and CS) and the number of copies of the α-*Gpdh^100^* allele and altitude in each sex were analysed using a Partial least square (PLS) analyses. This multivariate modelling analysis describes the relationship between sets of dependent (*partial warp scores*) and predictor variables (*genetic variation*, *altitude* and *CS*). The analyses were carried out separately for the two sexes in the SIMCA-S version 6.01 program [54].

Distribution pattern of the ecotypes were calculated by the posterior probability, based on the overall shape (*partial warp scores*), that a selected specimen of an ecotype belongs to a particular group (*sample localities/altitudinal level*). We thus use the over-all shape variation to estimate the number of montane ecotypes present at the different altitude levels rather than a visual determination. This analysis is done with a discriminate function analysis (DFA) and subsequent classification analysis. We expect to find specimens that are “misplaced” between ecotypes along the altitudinal gradient as also observed by Eriksson [28].

### Allozyme genotyping

Starch gel electrophoresis was used to measure genetic variation. A total of 14 enzyme systems were screened, of which nine coded for ten scorable loci; Adenylate kinase (*Ak*), Esterase (*Est-1* & *Est-2*), α-Glycerophosphate dehydrogenase (*α-Gpdh*), Hexokinase (*Hk*), Isocitrate dehydrogenase (*Idh*), Malate dehydrogenase (*Mdh*), Malic enzyme (*Me*), Mannose reductase (*Mnr*) and Triosephosphate isomerase (*Tpi*). Staining recipes and gel buffers are modified from those of Shaw & Prasad [55]. Starch gel electrophoresis, preparation of the beetles and allele classification follow Drotz *et al.* [21]. Enzyme systems where chosen to produce as high genetic variation as possible. Reproductive cross-experiments have verified their heritability, allele numbers and interpretability [56].

### Genetic analyses

Prior to analyses of population structure, we tested whether the assayed loci could be assumed to behave in a selectively neutral manner. Neutrality can be assumed if the confidence intervals of *F*_IP_ (the correlation of genes within Individuals relative to Population) overlap or lie between the confidence intervals of *F*_IP_ calculated over loci [57]. The over-population confidence intervals of *F*_IP_ were estimated from 5000 bootstrap randomisations. Per locus confidence intervals of *F*_IP_ were calculated from jackknife re-sampling. Genetic diversity of each locus over population was calculated using Nei's unbiased estimator [58] in the FSTAT v2.93 software [59].

In order to access the genetic differentiation of neutral markers between populations above and below the tree line, both within and between mountains, we utilise a three-level hierarchical analyses of gene frequencies. The strength of the population differentiation was estimated via pairwise *F*_ST_ calculations between populations at different elevation levels in mountain regions.

The relative levels of differentiation within and among altitudinal gradients in the mountain region (MR, areas D-J) were estimated from three-level hierarchical analyses of gene frequencies [60]. The following variance components were calculated from a three-level nested ANOVA: total variance 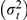, among areas 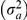, among populations within areas 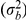, among individuals within populations 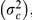 and within individuals (error term, 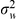). The estimators of genetic differentiation were obtained from these components as: 
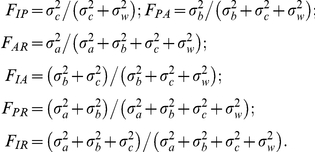

*F*_ip_ is the correlation of genes within Individuals relative to Population, *F*_pa_ the correlation of genes within Population relative to Areas, *F*_at_ the correlation of genes within Areas relative to Total, *F*_ia_ the correlation of genes within Individuals relative to Areas, *F*_pt_ the correlation of genes within Populations relative to Total, and *F*_it_ the correlation of genes within Individuals relative to Total. The analysis was carried out using SAS v8.1 software with the “NESTED” procedure. Ratios of sums were used to obtain per locus and overall estimations [61]. Both the *Mdh* and *Mnr* loci were excluded from this analysis since scores from the *Mdh* locus in Braket-XIV and from the *Mnr* locus in Gieravardo and Kråkberget-IV were not considered to be reliable.

To examine levels of genetic differentiation between elevations in the mountain region, population samples were designated as above (MA), at (MT) and below (MB) the tree line (Table 1). Genetic differentiation among populations within and between areas were estimated with pairwise *F*_ST_ at their respective elevations; calculated groups are MAxMA, MTxMT, MBxMB, MAxMT, MBxMT, and MAxMB. Population differentiation is expected to be higher among populations above the tree line (MAxMA) than among populations below and at the tree line (MTxMT, MBxMB), since the populations of flying forms should be more connected than the populations of non-flying forms. For similar reasons, genetic differentiation between MBxMT elevation levels should be lower than between MAxMT and MAxMB within mountains. Estimates were carried out according to Weir and Cockerham [61] and analysed in the FSTAT v2.93 software [59].

To estimate the impact of individual loci on the genetic differentiation between subsamples of ecotypes on either side of the tree line population structure was estimated with multivariate ordination of subpopulations by principle component analysis (PCA) of allele frequencies with the PCAGEN software [62]. This analysis is independent of the assumption of Hardy–Weinberg equilibrium. From the PCA analysis, a two-dimensional canonical plot of the first two principal components was produced. The PCA result is used to discriminate between site-specific (nonallopatric) and allopatric origin of divergence.This was done on the total material (not including the *α-Gpdh* loci) and independently for the mountain areas F to J where the population sample-size made the comparison statistically meaningful (Table 1). The purpose of this latter analysis was to test the prediction that if the two ecotypes have evolved in situ at each site, then the actual alleles contributing to the differentiation between ecotypes should be specific to each tree-line.

## Results

### Ecotype distribution

**Figure 3:**
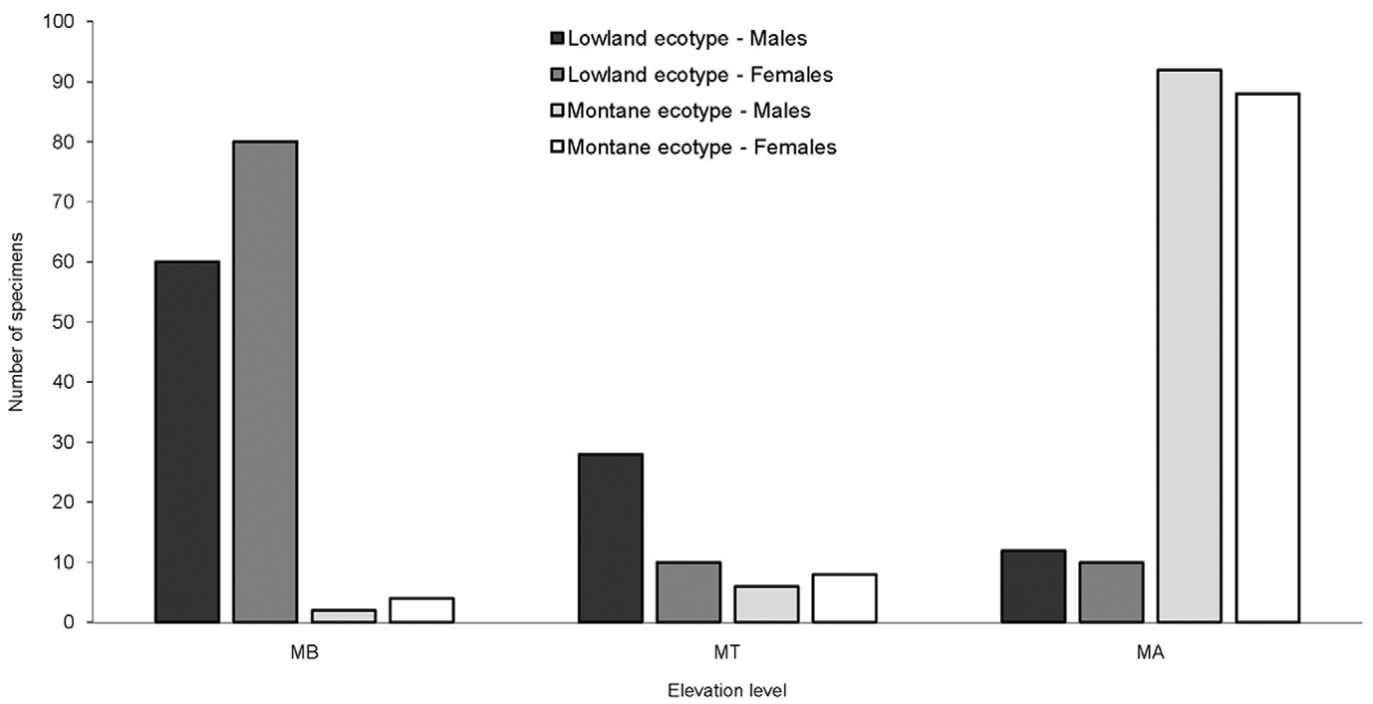
Location of *Agabus bipustulatus* ecotypes in relation to sampled area and elevation in northern Sweden. Morphological data were generated from spatial covariation of homologous landmarks that calculate affine (*partial warps*) and nonaffine (*uniform*) shape components as described by Bookstein [48]. Dispersal patterns implied via a reclassification model following a discriminate function analysis (DFA) of the shape parameters are as follows: Lowland ecotype – Male (▪); Lowland ecotype – Female (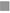); Montane ecotype – Male (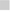); Montane ecotype – Female (□).

The shape variation described by the first five relative warps in both sexes visualised the shape difference between *A. bipustulatus* lowland and mountain ecotypes (Fig. 1) and combined explained 85.3% and 84.5% of the total variation for females and males, respectively. Significant morphological discrimination (partial warp scores) between ecotypes was detected within the mountain region elevation levels (Males: Wilk's lambda = 0.009, p<0.001; Females: Wilk's lambda = 0.008, p<0.001). The morphological variation was strongly influenced by elevation level, which explained 89% of the total variation in the females and 88% in the males. If all single specimens were reclassified according to elevation level and significant discriminate function 83% of the females and 71% of the males were reassigned to their correct elevation group. Both ecotypes showed a similar pattern of reassignment (Fig. 3). The lowland ecotype specimens were “misplaced” between elevation levels (MA or MB) approximately three to four times more often than the montane ecotype.

### Variation at the α-Gpdh locus

**Figure 4:**
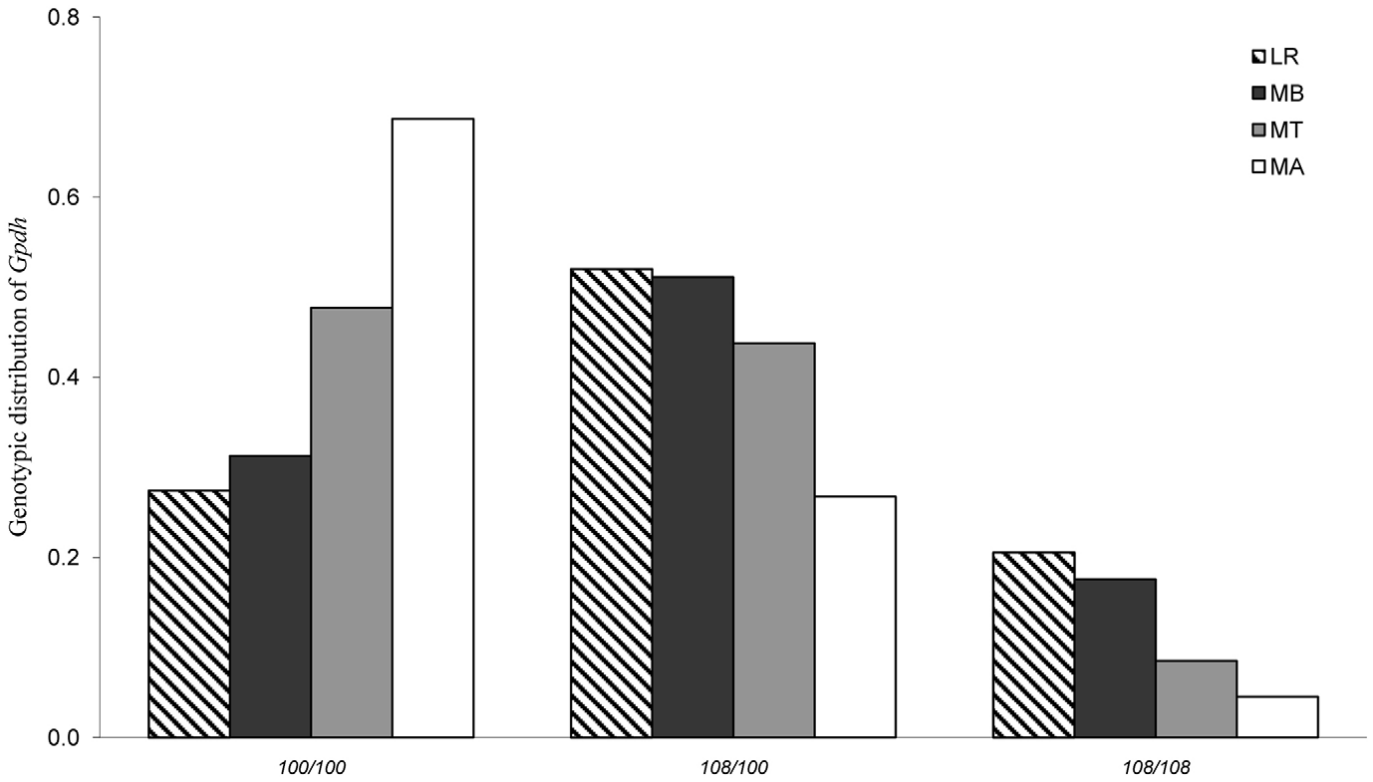
Genotypic frequencies of the *α-Gpdh* locus in *Agabus bipustulatus* in relation to elevation in northern Sweden. The ratio of sum over localities was used to obtain the genotype and overall estimates of genotypic frequencies. Lowland region (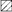); mountain region below tree line (▪), at the tree line (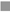) and above (□).

All loci surveyed, except *α-Gpdh* locus, had a none - significant overall population confidence interval (C.I.) of *F*_IP_ that overlapped the C. I. over loci. The *α-Gpdh* locus has two alleles named 100 and 108. The *α-Gpdh^100/100^* genotype is significantly more frequent in populations above than at or below the tree line in the altitudinal gradient (Kruskal-Wallis test; H = 8.78, P = 0.032). The frequency of the *α-Gpdh^108/100^* genotype, on the other hand, decreases successively from below, to at and above the tree line (Fig. 4). No significant differences were observed between all three genotypes below the tree line (MB) and populations in the lowland region (LR). The unbiased genetic diversity estimator (*H*) of the *α-Gpdh* locus can be roughly divided into three categories: 1) genetic diversity within populations below the tree line (MB) and in the lowland (LR) varies between *H* = 0.4–0.5; 2) populations at the tree line (MT) *H* = 0.2–0.3 and 3) populations above the tree line (MA) *H*<0.1. However, exceptions exist; Rörmyrberget-III in the lowland has a genetic diversity of *H* = 0.32, and a few populations at the tree line in area G, I and J have a genetic diversity of 0.39 to 0.50, and no differences were observed across the tree line in area F and from the tree line to above in area E. Genotypic frequencies for each sampled population is given table 1.

### Covariation of morphology and α-Gpdh across the tree line

**Figure 5:**
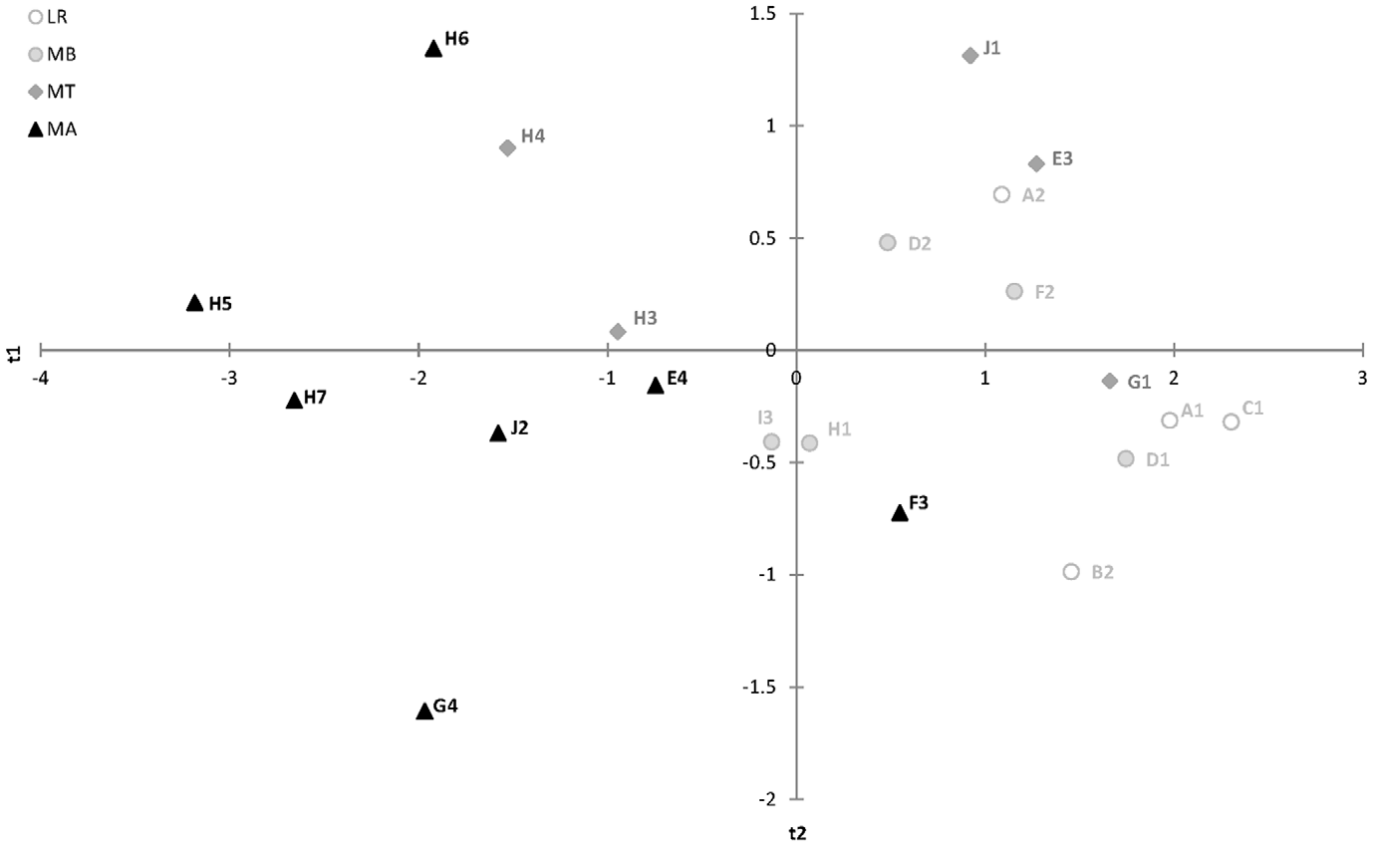
Morphological relationship between the *α-Gpdh* ^100^ allele and altitude in female *Agabus bipustulatus*. *Agabus bipustulatus* female score plot from the PLS analysis of the linear relationship between the first significant (R1) component between morphology (*partial warp*) and the mean number of copies of the *α-Gpdh* ^100^ allele per populations, altitude and beetles size (*CS*). The correlation between the Y- and X-matrix was 0.70. Populations from lowland region (○); mountain region below tree line (•), at the tree line (▴) and above (▪). Population numbers are given according to table 1.

The relationship between morphological variation (partial warp scores), altitude, CS (size) and the number of copies of the α-*Gpdh* ^100^ allele per population, were estimated with the Partial least square (PLS) analysis for both sexes independently. The analyses resulted in one significant (R1) component for each sex, with a correlation of 0.63 for females and 0.52 for males (Fig. 5). The significant components explained 17.6% of the morphological variation and 39.8% of the genetic variation in the α-*Gpdh* locus for females and 14.8% and 26.9% respectively for males. The predictive power (explained variation) of the PLS models for the female and male analyses was 36.4% and 14.3%, respectively. The highest loading values were observed for the number of copies of the α-*Gpdh* ^100^ allele and altitude in both sexes.

### Genetic structure

The genetic diversity over all populations was normally distributed and varied from 0.153 to 0.404 with an overall mean and standard deviation (SD) of 0.261±.07. Below the tree line, genetic diversity was similar in both the LR and MR. At the tree line, genetic diversity was similar in both the LR and MB regions. The diversity increased slightly at the tree line and then decreased moderately above it but these differences are not significant (Kruskal-Wallis test; H = 5.384, P = 0.146).

Significant positive deviations in genetic expectations under random mating were observed within 55% of the populations, as seen in the *F*_ip_ values per population (Table 1). The *F*_ip_ values were normally distributed and varied between −0.032–0.449 over all populations (between −0.032–0.407 in the lowland region and 0.059–0.449 in the mountain region), with a mean and standard deviation of 0.203±0.12.

### Local genetic differentiation

In the mountain region, genetic differentiation between populations within areas and between populations within the region showed a moderate level in the hierarchical analysis, *F*_pa_ = 0.080 and *F*_pt_ = 0.075, respectively (Table 2). Most genetic variance is found among individuals within populations, 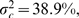 and 10.1% was found among populations within areas 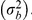 Genetic differentiation was not detected between areas (*F*_at_ = 0.0 and 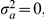 respectively). Pairwise *F*_st_ calculations, not including the *α-Gpdh* locus, were used to estimate genetic differentiation within and between different mountain areas, and between the three elevation classes: below (MB), at (MT) and above (MA) the tree line. These calculations showed that the mean pairwise *F*_st_ values between the elevation classes are higher between than within mountain areas. For elevation classes within mountain areas, the genetic differentiation increases from *F*_st_ = 0.026, between MB and MT, to *F*_st_ = 0.071 between MT and MA (Table 3). The increase is less dramatic when elevation classes were compared between mountain areas, MBxMT, *F*_st_ = 0.051 and MTxMA, *F*_st_ = 0.078. A similar pattern of differentiation is also seen when elevation classes MB and MA are compared within and between areas, *F*_st_ = 0.065 and 0.075, respectively. Genetic differentiation among populations below and at the tree line is similar within mountains in the mountain region, whereas the genetic differentiation is always higher among populations when the montane ecotype above the tree line is included in comparisons, thus *F*_st_ = 0.050 within and 0.062 between mountains.

**Table 2:** Estimated variance components and three-level hierarchical F-statistics of the genetic variation of Agabus bipustulatus.

Locus	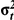	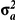	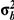	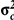	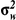	*F*_ip_	*F*_pa_	*F*_at_	*F*_ia_	*F*_pt_	*F*_it_
*Tpi*	0.01502	0.00007	0.00057	−0.00080	0.01437	−0.0590	0.0403	0.0049	−0.0163	0.0450	−0.0113
*Me*	0.50087	0.00049	0.01454	0.18816	0.29594	0.3887	0.0292	0.0010	0.4065	0.0301	0.4071
*Idh*	0.50030	0.00167	0.01442	0.05706	0.42655	0.1180	0.0290	0.0033	0.1435	0.0322	0.1464
*Ak*	0.00364	−0.00001	0.00005	−0.00004	0.00359	−0.0113	0.0139	−0.0028	0.0028	0.0111	0.0000
*Hk*	0.50000	0.00371	0.03058	0.20351	0.26220	0.4370	0.0616	0.0074	0.4717	0.0686	0.4756
*Est-1*	0.50852	−0.01660	0.10129	0.30093	0.10587	0.7397	0.1994	−0.0338	0.7916	0.1723	0.7846
*Est-2*	0.50747	−0.01433	0.09469	0.23790	0.17426	0.5772	0.1868	−0.0291	0.6562	0.1632	0.6462
Overall	0.50349	–	0.05086	0.19591	0.25470	0.3129	0.0800	−0.0070	0.3509	0.0746	0.3498
Present *%*			10.1	38.9	50.6						

Estimated variance components within a mountain region in northern Sweden; total variance 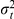, among regions 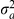, localities within region 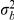, individuals within localities 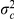 and within individuals 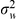. F-statistics abbreviations individual (I), population (P), area (A), and total (T).

**Table 3:** Mean pairwise FST estimates between populations at altitudinal elevations of Agabus bipustulatus.

Elevation level comparison	Within areas		Between areas	
	Mean *F*_ST_	Std. err.	Mean *F*_ST_	Std. err.
MB×MB	0.040	0.020	0.056	0.011
MB×MT	0.026	0.010	0.051	0.006
MB×MA	0.065	0.019	0.075	0.008
MT×MT	0.039	0.013	0.042	0.008
MT×MA	0.071	0.013	0.078	0.008
MA×MA	0.050	0.009	0.062	0.012

Mean pairwise FST estimates (and their standard error) between populations of Agabus bipustulatus at different altitudinal elevations, within and between mountain areas in northern Sweden. Populations at (MT), above (MA), and below (MB) the tree line.

**Figure 6:**
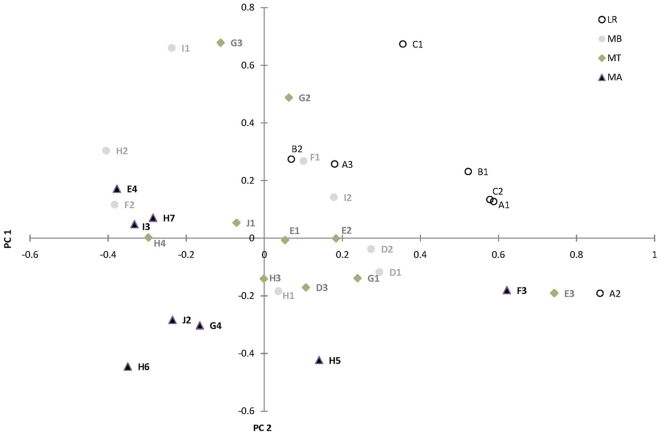
Genetic relationship between *Agabus bipustulatus* populations and altitudinal levels in northern Sweden. Genetic relationship was analysed from nine enzyme systems (not including the *α-Gpdh* loci) with a principle component analysis (PCA). The first significant component from the PCA separated the two ecotypes with an overlap of the populations at the tree line and explained 36.0% of the total genetic variation. The second significant axis of the PCA explained 17% of the genetic variation. Populations from lowland region (○); mountain region below tree line (•), at the tree line (▴) and above (▪). The deviating population above the tree line to the right corner is Gäutavardo (F3) from the F area in the mountain region. Population numbers are given according to table 1.

These results are congruent with the Principal components analysis (PCA) of the population structure, which explained 53% of the total genetic variation (not including the α-*Gpdh* locus) with a global *F*_st_ value of 0.097 (Fig. 6). Allele frequencies along the first significant component separated the two ecotypes with an overlap of the populations at the tree line, explained 36% of the genetic variation. The second axis was significant and explained 17% of the variation. The gradual genetic change is very strikingly but some population stands out, e.g. Gäutavardo (F3) and Kråkberget-VI (E3), as being more similar to the genetic composition of their ecotype counterpart.

The five loci with highest impacts (loading scores) separating the two ecotypes in the PCA analyses of mountains F to J are, in descending order: Area ***F:*** *Ak*, *Mdh*, *Mnr*, *IDH*, *Est-2*; ***G:*** *IDH*, *Est-1*, *Est-2*, *Me*, *Mdh*; ***H:*** *Ak*, *IDH*, *Mdh*, *Mnr*, *Est-2*; ***I:*** *Ak*, *HK*, *Me*, *Mdh*, *Mnr*, and area ***J:*** *Ak*, *IDH*, *Est-1*, *Mdh*, *Mnr*. Clearly, the alleles and loci that reflected ecotype separation in any one mountain area differed from the alleles and loci that separated the ecotypes in the other areas. This is strong support for *in situ* evolution of ecotypes within each mountain area.

## Discussion

Here we will argue that the divergence between *A. bipustulatus* lowland and montane ecotypes in morphology and α-*Gpdh* genotype reflects independent local adaptive differentiation *in situ* across altitudinal gradients via four main lines of evidence: (1) Repeated patterns of morphological differentiation are observed in all independent gradients across the tree line, with high reclassification probabilities, at elevation levels (MA, MT and MB). Minor admixing of ecotypes occurs at all altitudinal levels. (2) The allelic and genotypic variation at the α-*Gpdh* locus is strongly correlated with altitude and habitats across the tree line, a pattern not observed in molecular markers deemed as neutral. (3) Molecular markers indicate that the strongest genetic differentiation is observed between ecotypes and that this differentiation is more pronounced between mountain areas than within, implying that gene flow may be impeded as a result of local adaptation. (4) The genetic differentiation between ecotypes, estimated from the enzyme systems, is reflected through different sets of loci in each independent mountain area. Evidently, the observed ecological transitions within *A. bipustulatus* across altitudinal gradients take place over short distances and are likely to be associated with variation in habitat-related fitness. Drotz *et al.* [23] have documented these associations between flightlessness and α-*Gpdh* variation in *A. bipustulatus* across the entire western Palaearctic region. The smaller scale process documented here could thus shed new light on the high morphological and taxonomic diversity within this species that has been discussed for more than 120 years [19], [21], [28].

### Flight muscle hydrolysis and dispersal

The interpretation of the morphological and genetic adaptation across the tree line will depend on whether the dispersal potential of the ecotypes exceeds the realised gene flow [10], [13]. Moreover the dispersal potential of the ecotypes will influence the cline width and the strength of the selective forces needed to maintain differentiation across the local hybrid zone [63].

Similar to the results of Eriksson [28], who showed that only 5.6% of 356 specimens of the mountain ecotype were found below the tree line in Finnish Lapland whereas 94% of the specimens above the tree line had reduced flight muscles, the morphological reclassification model, in this study, found higher numbers of “misplaced” specimens between below and at the tree line than above it (Fig. 3). This indicates that movement of beetles within valley floors are more common than between mountain tops of different Valley floors, while the downward dispersal of the montane ecotype is about 3–4 times lower than the upward migration of the lowland ecotype.

A comparison of the upward and downward migration may shed some light on when the flight muscle histolysis occurs. If muscle autolysis, which is a common phenomenon within Coleoptera [64], occurs prior to oviposition or in the pupal stage, dispersal is more or less geographically restricted within water systems [63]. This may be true for *A. bipustulatus*. If hydrolysis begins after oviposition we would observe more montane type specimens below the tree lines in the mountain region, since more specimens should be able to fly. Our results indicate that the hydrolysis starts prior or during the pupal stage. Dispersal of the mountain ecotype should be more or less restricted to short dispersal events within lakes and/or downstream movements within water systems. These assumptions are supported by the morphological data for e.g. Atoklinten-II (H5), Atoklinten-IV (H7) and Braket-XIV (J2) which consist of individuals of extreme mountain type, high number of copies of the α-*Gpdh* ^100^ allele and come from different mountain ridges above the tree line (Fig. 5). These observations are interesting, since Atoklinten-I–III (H3, H5–6) is genetically closer to each other than to Atoklinten-V (H4) and Atoklinten-IV (H7) which belong to different water systems. This genetic similarity is also seen between Braket-XIV (J2) and Lillbraket (J1) which are only 1 km apart on the same mountain slope and share the same water system (Fig. 6). In contrast, to Djuptärn (I3) and Kråkberget-VII (E4) which originate from the same altitude, but consist of less extreme specimens of the mountain type or are intermixed with the lowland type. They have a lower frequency of the α-*Gpdh* ^100^ allele. Gäutavardo (F3) is more similar to the lowland type morphological and genetically than to the mountain type (Fig. 6). Dispersal from the valley floor upwards across the tree line is therefore assumed to be mainly done by active flight.

### Genetic structure

Large significantly positive *F*_ip_ values were observed in most samples of both ecotypes of *A. bipustulatus*. These values are within the range observed in other water beetles, e.g. [22], [56], [65]. A reasonable explanation for this is common short-range dispersal behaviour of water beetles. Using marked beetles, both Süselbeck [34] and Davy-Bowker [38] showed that the autumn increases and spring decreases in population sizes are at least in part due to adults emerging from the pupae stage, immigration and emigration, respectively. They also noted that the beetles often only made one or few long distance migration bouts during the early stage of their life cycle after which they tend to stay in one specific region within a lake or smaller pond [29], [34]. Specimens of *A. bipustulatus* tend to aggregate near large stones, where shore vegetation creates cover, or under large rocks near the shore line. However, beetles can be found all over the water body and bottom areas in smaller lakes. There is therefore a higher probability to sample different “family” aggregates due to admixture in larger habitats. This effect might explain the higher mean population *F*_ip_ values [66] above the tree line in relation to other altitudinal levels and the lowland region, since the habitats above the tree line are larger than at or below it. Only two out of 13 smaller populations with fewer than 15 specimens had a significant *F*_ip_ deviation (Table 1).

### Adaptation across the tree line

The distance between populations below, at and above the tree line within each mountain area is short and well within the dispersal range of water beetles [29]. In spite of this, large genetic and morphological differences were detected between populations. Different neutral enzyme systems reflected these changes between ecotypes on the different mountains (Fig. 6). These observations indicate a constraint to gene flow and an independent adaptation of populations above the tree line (MA). This is supported by the variance components analysis (Table 2) that showed that the genetic variance was higher within populations and mountain areas across the tree line than between collecting areas.

Levels of differentiation between populations in the lowland region (LR) and below the tree line (MB) in the mountain region (MR) are of similar magnitude implying similar levels of connectedness (Fig. 6). Gene flow within the mountain region, implied by pairwise *F*_st_, takes place more easily between stable and temporary habitats along the Valley floor (MB) up to the tree line (MT) than between areas (Table 3). Genetic differentiation between populations across the tree line increases by a factor of three within areas and by 1.5 between areas in the mountain region. The estimated contribution of gene flow, not including the *α-Gpdh* locus, is therefore more pronouned from the Vally floor up past the tree line (Fig. 6). However, at the tree line populations tend also morphologically to be a mixture of specimens that are either of one or the other ecotype intermixed with few specimens of the other type (Fig. 5). The adaptive divergence consequently is most profound in populations above the tree line (MA). Gene flow does not, however, manage to conteract the effect of selection [8]–[9] and the morphological transition across the tree line must be abrupt, since few lowland ecotype specimens are found above the tree line (Fig. 3). The selection acting directly or indirectly on the *α-Gpdh^108^* allele must be strong [12], [63], [67]–[68].

Studies of wing dimorphic species or comparisons between flying and non-flying species have often shown extensive genetic differentiation between populations or species. Within the flightless stream dwelling waterstrider *Aquarius remigis*, *F*_st_ values of 0.46 are reported among streams [69]. Lower mean heterozygosity values are reported for non-flying than flying waterstriders, *H* = 0.058 and 0.234, respectively [70]. Large *F*_st_ values have also been reported between flying and non-flying carabid species, *F*_st_ = 0.003–0.16 and 0.26–0.27, respectively [71]–[72]. A conclusion drawn from these studies is that population differentiation increases with reduced dispersal potential. Such a pattern supports our argument that flight muscle hydrolysis occurs prior to or during the pupal stage.

In conclusion, there is ample evidence that adaptive divergence maintains local ecotypic differentiation in spite of ongoing gene flow in *A. bipustulatus*. Thus the *A. bipustulatus* complex emerges as a promising example of non-allopatric evolution of *in situ* reproductive isolation [4]–[6], [14]–[16]. In a future perspective we may ask the following question: is the morphological transition reversible between the parental generation and offspring? Is it possible to regulate/initiate the morphological transformation between ecotypes with temperature? Could this adaptive process described here explain the evolution of many high altitudinal water beetle species within the Palaearctic region [68]?
